# Epigenetic reprogramming of NK cell-mediated antitumor immunity in urothelial carcinoma: a mini-review

**DOI:** 10.3389/ebm.2026.11373

**Published:** 2026-10-28

**Authors:** Himani Kumari, Chih-Chieh Chou, Yu-Ting Lee, Cheng-Huang Shen, Michael W. Y. Chan

**Affiliations:** 1 Department of Biomedical Sciences, National Chung Cheng University, Chia-Yi, Taiwan; 2 Epigenomics and Human Diseases Research Center, National Chung Cheng University, Chia-Yi, Taiwan; 3 Division of Hematology and Oncology, Ditmanson Medical Foundation Chiayi Christian Hospital, Chiayi, Taiwan; 4 Department of Urology, Ditmanson Medical Foundation Chiayi Christian Hospital, Chiayi, Taiwan; 5 Research Center for Precision Environmental Medicine, Kaohsiung Medical University, Kaohsiung, Taiwan

**Keywords:** epigenetics, immunotherapy, natural killer cell, NKG2D, urothelial carcinoma

## Abstract

Urothelial carcinoma (UC) is a dynamic malignancy characterized by a complex epigenetic landscape. Epigenetic modifications are pivotal in modulating the innate immune response, particularly within the tumor microenvironment (TME), where they profoundly influence natural killer (NK) cell function. Aberrant epigenetic regulation often promotes immune evasion by silencing activating ligands on tumor cells, thereby impairing NK cell-mediated cytotoxicity. Emerging epigenetic therapies, such as histone deacetylase inhibitors (HDACis) and DNA methyltransferase inhibitors (DNMTis), offer significant potential to reprogram the immune landscape and restore anti-tumor immunity. Despite this promise, clinical implementation faces challenges, including the immunosuppressive nature of the TME, NK cell exhaustion, and complex manufacturing limitations. Combining epigenetic agents with NK cell-based immunotherapies represents a robust strategy to overcome these barriers and improve therapeutic outcomes. This review elucidates the epigenetic regulatory mechanisms in UC and emphasizes the necessity of personalized, biomarker-driven treatment strategies to optimize cancer immunotherapy outcomes.

## Impact statement

Urothelial carcinoma (UC) represents a highly adaptive malignancy with substantial epigenetic underpinnings that critically influence immune surveillance. In this mini-review, we explore the intersection of epigenetic regulation and innate immune modulation, focusing particularly on natural killer (NK) cells within the tumor microenvironment (TME). We highlight how epigenetic alterations contribute to immune evasion by tumor cells, and we assess the current and emerging roles of epigenetic therapies, including histone deacetylase inhibitors (HDACis) and DNA methyltransferase inhibitors (DNMTis), in restoring NK cell-mediated cytotoxicity. Given the increasing relevance of epigenetic therapy and innate immunity in cancer research, we believe this review will be of high interest to the readership of Cancer Biology section of EBM. It provides a timely synthesis of the current landscape and outlines a framework for future investigation and therapeutic innovation.

## Introduction

Urothelial carcinoma (UC) is a clinically and molecularly heterogeneous disease. While its etiology is often linked to environmental factors, such as tobacco smoke, industrial aromatic amines, or arsenic exposure, its clinical trajectory is shaped by a complex interplay of anatomical features, tumor-intrinsic biology, host factors, and treatment-related variables [1]. The disease typically affects older adults with a male predominance, and while the majority of cases arise in the bladder, upper tract urothelial carcinoma (UTUC) remains a formidable diagnostic and prognostic challenge [2, 3].

In clinical practice, the management of UC is dictated by the anatomical boundary of the detrusor muscle. This threshold separates Non-Muscle-Invasive Bladder Cancer (NMIBC) from Muscle-Invasive Bladder Cancer (MIBC), serving as the primary determinant for therapeutic intervention [4].

For NMIBC (Ta, T1), the goal is bladder preservation. Standard treatment involves transurethral resection of bladder tumor (TURBT), followed by intravesical therapies. *Bacillus* Calmette–Guérin (BCG) remains the gold standard for intermediate- and high-risk patients, while intravesical chemotherapy is used for patients with lower-risk disease [2]. However, the clinical community faces a “BCG-unresponsive” crisis, necessitating urgent research into novel intravesical agents and systemic immune primers [5].

Once the tumor breaches the muscle (≥T2), the disease requires a systemic, multimodal approach. This typically involves cisplatin-based neoadjuvant chemotherapy, designed to eradicate micro-metastases, followed by radical cystectomy and pelvic lymph node dissection [3].

The therapeutic landscape for advanced and metastatic UC has evolved significantly over the last 3 decades. Platinum-based chemotherapy is no longer the sole treatment modality, the toolkit now includes immune checkpoint inhibitors (ICIs), such as pembrolizumab and nivolumab, and the avelumab maintenance therapy, which has become a standard approach for prolonging survival [6]. Recent advances in precision medicine have introduced “smart” therapies. The approval of erdafitinib for patients with FGFR2/3 alterations marked a critical shift toward biomarker-guided care [7]. Simultaneously, antibody-drug conjugates (ADCs) like enfortumab vedotin are redefining expectations, particularly when utilized in combination with ICIs [8].

## The molecular engine: epigenetics and immunity

Underpinning these clinical shifts is a complicated molecular landscape. Large-scale sequencing, such as the TCGA, has revealed that MIBC is profoundly shaped by epigenetic dysregulation. Recurrent mutations in chromatin remodeling and histone-modifying genes, such as *ARID1A*, *KDM6A*, *CREBBP/EP300*, and *SMARCB1*, coexist with alterations in the PI3K/AKT/mTOR and DNA damage repair pathways [9, 10]. These mutations can disrupt chromatin accessibility and transcriptional regulation, thereby affecting pathways involved in tumor suppression, DNA repair, and immune responses. For example, loss of ARID1A alters immune-related transcription and DNA repair, while KDM6A mutations are also frequent epigenetic alterations in bladder cancer [9, 10].

These alterations fundamentally reshape how the tumor interacts with the immune system. This “epigenetic reprogramming” creates distinct molecular subtypes with differing therapeutic vulnerabilities [11]. Consequently, understanding how these epigenetic shifts orchestrate the anti-tumor immunity is essential for moving beyond “one-size-fits-all” surgery toward precision immunotherapy [12]. Epigenetic dysregulation further contributes to UC development and progression. Promoter DNA hypermethylation, such as *ZNF671, IRF6, RARB2, GSTP1*, together with alterations in repressive histone marks, including H3K27me3, and H3K9me3, may suppress tumor suppressor genes and modify chromatin accessibility [13, 14]. These molecular alterations may also have clinical relevance. For example, ZNF671 promoter methylation has been associated with an increased risk of recurrence, whereas mutations in the chromatin-remodeling gene ARID1A have been proposed as potential biomarkers of response to immune checkpoint inhibitors [9, 15].

Beyond regulating tumor-intrinsic properties, genetic and epigenetic alterations can influence cytokine signaling, antigen presentation, and immune-cell recruitment, thereby shaping interactions between UC cells and their surrounding microenvironment [16]. The UC tumor microenvironment (TME) is highly heterogeneous, comprising immune cells, stromal cells, blood vessels, and extracellular matrix. Understanding the TME is essential not only for unraveling tumor biology but also for harnessing this information to refine precision therapy strategies [17].

## Epigenetic dysregulation in urothelial carcinoma

Urothelial carcinoma is frequently defined as an epigenetic disease. Whole exome sequencing has revealed frequent alterations in genes responsible for chromatin organization and histone modifications, either through mutation or altered expression [13]. The basic structural unit of chromatin is the nucleosome, in which negatively charged DNA wraps around positively charged histones. Modifications to this structure, alongside DNA methylation, drive epigenetic silencing. For example, DNA methylation at promoter CpG islands often suppresses gene transcription and serves as a promising biomarker for cancer detection in urine samples, specifically regarding genes like *ZNF671*, *IRF6*, and *GSTP1* [18].

Mutations in chromatin-regulating proteins, such as *ARID1A* [9], *IDH1/2*, *TET2*, and *DNMT3A* [7], significantly disrupt the epigenome. These mutations alter DNA and histone methylation processes, contributing to various malignancies, including gliomas and acute myeloid leukemia (AML), as well as UC [10]. Histone post-translational modifications (PTMs), such as acetylation, methylation, ubiquitination, and phosphorylation, further modulate cellular processes, including DNA repair and differentiation [19]. Active histone marks, such as H3K27ac and H3K4me3, are associated with transcriptional activation [20], whereas repressive marks, like H3K9me3 and H3K27me3, are linked to gene silencing and are heavily implicated in UC progression [14].

MicroRNAs (miRNAs) represent another layer of regulation, post-transcriptionally modulate gene expression by binding to the 3′UTR of target mRNAs, leading to mRNA degradation or translational inhibition [21]. Given these mechanisms, targeting epigenetic modifiers offers the potential to reprogram the immune landscape and enhance anti-tumor immunity, highlighting the critical role of epigenetics in advancing cancer immunotherapy [22].

## Epigenetic regulation in NK-cell mediated immune evasion

Tumor immunosurveillance refers to the ability of the immune system to recognize and eliminate transformed cells. However, clinically detectable tumors may have acquired mechanisms that enable them to evade immune control, corresponding to the “escape” phase of cancer immunoediting [23]. Although their role in tumor immunosurveillance has historically received less attention than that of T cells, NK cells are increasingly recognized as critical antitumor effectors because they can detect and eliminate transformed cells without prior antigen priming [24].

Within the innate immune response, NK cells (characterized as CD56^+^, CD16^+^ and CD3^−^) serve as a frontline defense against malignant cells, capable of exerting cytotoxic effects without prior sensitization [25–28]. NK cells eliminate tumor cells through direct lysis or by secreting interferon-gamma (IFN-γ) [29]. Their activity is tightly regulated by the balance between activating and inhibitory receptors [30]. Among these, the activating receptor NKG2D (natural-killer group 2, member D), plays a pivotal role in immune surveillance by recognizing stress-induced ligands (NKG2DLs), including UL16-binding proteins (ULBPs) and MHC class I chain-related proteins A and B (MICA/B) [31, 32]. Engagement of NKG2D triggers DAP10-dependent signaling, leading to the recruitment of downstream effectors, including PI3K and Grb2-Vav1, and subsequent cytoskeletal reorganization and polarization of cytotoxic granules toward the immunological synapse. Subsequent granule exocytosis releases perforin and granzyme B, leading to tumor-cell killing [31, 33]. However, cancer cells frequently evade this surveillance by downregulating NKG2DLs, an event often mediated by the hostile TME and complex epigenetic changes [34, 35].

In UC, the interplay between tumor cells and NK cells is profoundly influenced by epigenetic alterations. Aberrant DNA methylation and histone modifications can suppress the expression of ULBPs and MICA/B, thereby promoting immune evasion. In parallel, epigenetic dysregulation within NK cells can impair their cytotoxic capacity through the suppression of key effector molecules, such as granzyme B and perforin [36]. At the molecular level, repression of MICA/B and ULBP1–6 can involve multiple epigenetic regulators and repressive chromatin mechanisms, including DNMT1/3A/3B, class I/II HDACs, and EZH2-associated H3K27me3 (Figure 1) [36].

**FIGURE 1:**
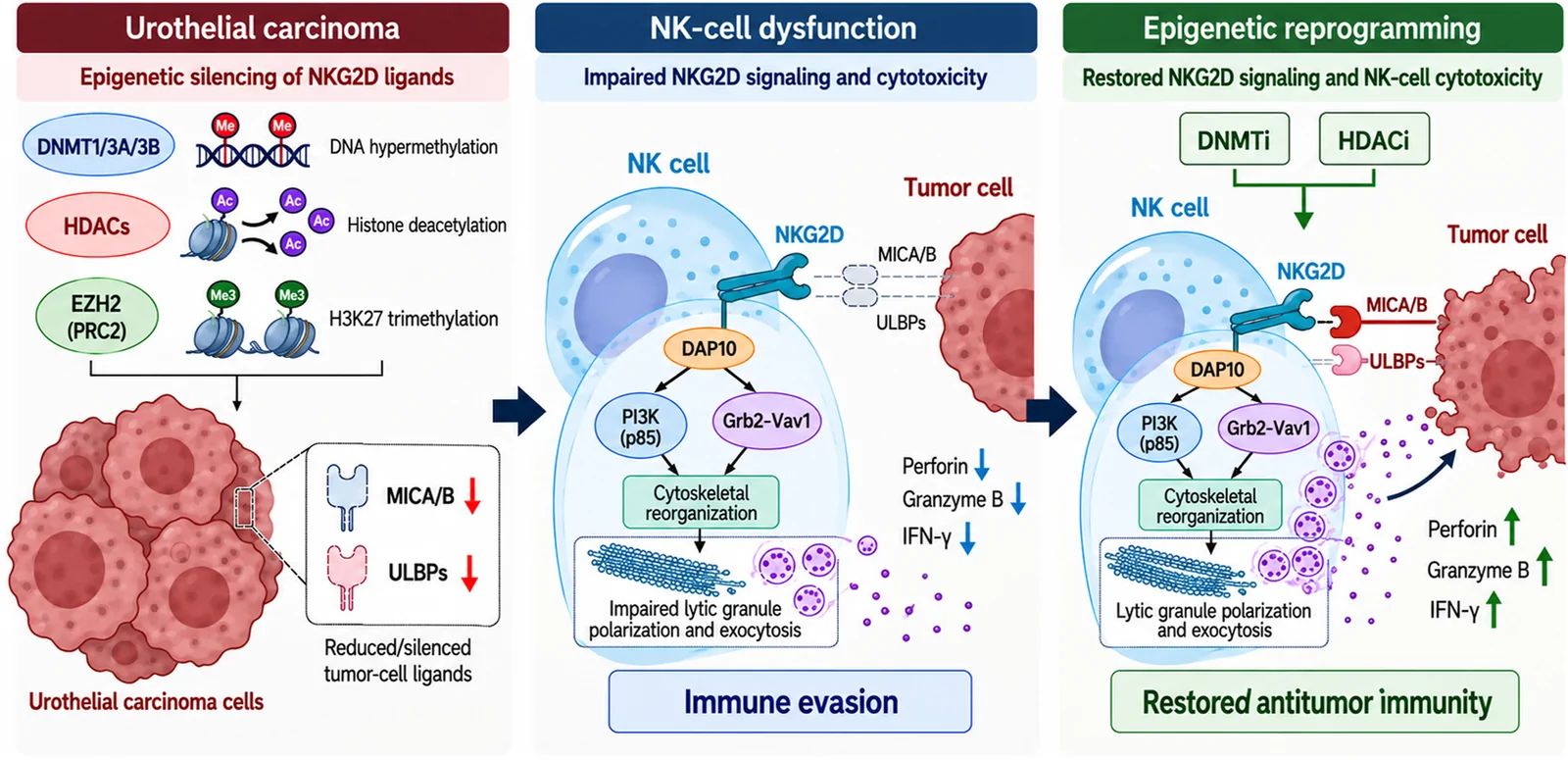
Epigenetic repression of MICA/B and ULBP1-6 in urothelial carcinoma. Promoter regions of MICA, MICB, and ULBP1–6 can undergo DNA hypermethylation mediated by DNA methyltransferases (DNMT1, DNMT3A, and DNMT3B), histone deacetylation catalyzed by class I/II histone deacetylases, and histone H3K27me3 catalyzed by EZH2 within the Polycomb repressive complex 2 (PRC2). These repressive epigenetic modifications lead to a more compact chromatin structure, reduced transcription of MICA/B and ULBP1-6, decreased cell surface expression of NKG2D ligands on urothelial carcinoma (UC) cells, and ultimately impaired recognition and killing by NK cells and CD8^+^ T cells.

Epigenetic therapies can reverse these escape mechanisms. DNMT inhibitors (e.g., decitabine, azacitidine) can demethylate promoters of NK-activating ligands, restoring their expression. HDAC inhibitors (e.g., entinostat, vorinostat) have been shown to upregulate NKG2D ligands and enhance NK-cell cytotoxicity. By reprogramming the TME, these agents sensitize UC tumors to immune checkpoint inhibitors, creating synergistic effects that position epigenetic modulation as a promising Frontier for personalized immunotherapy [22].

## Clinical implications of epigenetic therapies

DNA methylation plays a pivotal role in regulating NK cell function within the TME. Aberrant methylation patterns can silence key activating receptors and effector genes in NK cells, impairing their cytotoxic potential [37]. Therapeutic demethylating agents such as decitabine have demonstrated the ability to restore NK cell function by reactivating silenced immune genes [38].

Similarly, histone modifications, particularly methylation and acetylation, are critical. Repressive marks like H3K27me3 have been associated with the downregulation of cytotoxic genes like perforin and granzyme B [39]. Previous studies have demonstrated that treatment with HDAC inhibitors, such as entinostat, enhances NK cell-mediated cytotoxicity by promoting the expression of epigenetically suppressed ULBPs and other NKG2DLs, as demonstrated in our recent publication [40].

Furthermore, RNA interference (RNAi) mechanisms contribute to the post-transcriptional regulation of NK cell function. For example, RNAi targeting adrenomedullin has been shown to reduce proliferation in bladder cancer cells, indirectly enhancing NK cell-mediated clearance [41]. Overall, the TME imposes immunosuppressive pressures reinforced by histone modifications and RNA interference, both contributing to transcriptional silencing and impaired NK cell cytotoxicity [42].

Beyond preclinical studies, epigenetic therapies have advanced into clinical evaluation. Early-phase (phase I/II) trials have investigated DNMTis such as decitabine and azacitidine, demonstrating their ability to demethylate tumor suppressor genes and restore immune recognition [43]. HDACis, including vorinostat and entinostat, have also been tested, showing potential to upregulate NK-cell activating ligands and sensitize UC tumors to immune checkpoint blockade [44]. While toxicity profiles were manageable, clinical efficacy was often limited when used as monotherapy, underscoring the need for rational combinations [45]. Ongoing Phase II/III trials are now evaluating DNMTis or HDACis alongside PD-1 inhibitors like pembrolizumab, aiming to overcome resistance and improve durable responses in advanced UC. These trials reflect a paradigm shift: epigenetic agents are being repositioned from cytotoxic modifiers to immune-sensitizing therapies capable of reprogramming the tumor microenvironment [46].

## Discussion

Despite the promise of NK cell-based immunotherapy, its effectiveness against solid tumors remains limited by the immunosuppressive TME, which impairs NK-cell infiltration, persistence, and cytotoxicity and promotes functional exhaustion [47, 48]. Strategies to overcome these challenges include the blockade of immune checkpoints, metabolic reprogramming of the TME, and the enhancement of NK cell survival within tumor sites [29, 49, 50]. A recent study further demonstrated that the BET family protein BRD4 cooperates with SMAD3 to promote the transcription of inhibitory receptors and immune checkpoints associated with NK-cell exhaustion. Accordingly, BET inhibition downregulated these inhibitory receptors and restored NK-cell cytotoxicity against non-small cell lung cancer [51].

As NK cell therapies advance, Good Manufacturing Practice (GMP) production presents significant challenges. Large-scale manufacturing with stringent quality control is essential for patient safety. Academic-industry partnerships are critical for bridging the gap in commercial production, necessitating advancements in cryopreservation and automation to produce off-the-shelf, cost-effective NK cell products [52].

In addition, engineered cell therapies, particularly chimeric antigen receptor (CAR)-NK cells, are emerging as a promising strategy for urological malignancies. Early-phase clinical studies of CAR-NK therapies are currently being pursued in other urological cancers, including CD70-targeted CAR-NK approaches in renal cell carcinoma. However, registered CAR-NK trials specifically for urothelial carcinoma remain lacking. In UC, tumor-associated surface antigens such as Nectin-4, HER2, EGFR, and CD24 represent potential targets for engineered cell therapies, with CD24-directed CAR-NK-92 cells already demonstrating preclinical antitumor activity in bladder cancer models [53].

Looking ahead, epigenetic modifications are expected to transform the therapeutic landscape of UC. First, DNA methylation and histone modification profiling will increasingly serve as biomarkers to stratify patients [54]. Second, the integration of epigenetic modulators with ICIs and ADCs could enhance durable responses in advanced UC [55]. Epigenetic modulation may also provide a complementary strategy to enhance CAR-NK therapy. DNMT and HDAC inhibitors can increase the expression of NK-cell activating ligands, including ULBPs and MICA/B, potentially improving tumor-cell recognition. Emerging evidence further indicates that HDAC inhibition can directly enhance CAR-NK function; entinostat was shown to sustain CAR expression and improve the persistence and antitumor activity of CAR-NK cells in preclinical models [56, 57]. Nevertheless, whether epigenetic preconditioning can overcome stromal barriers and improve CAR-NK homing, infiltration, and persistence specifically in UC remains to be established.

Finally, clinical trial design must evolve to integrate epigenetic agents with NK cell-based immunotherapies. Expanded summary tables and figures, detailing UC-specific epigenetic alterations, NK-cell pathways, and therapeutic implications, will be critical for guiding translational research and clinical application [18].

This review highlights the pivotal role of epigenetic modifications in regulating the innate immune response, particularly in urothelial carcinoma (Figure 2). Epigenetic changes, including DNA methylation and histone modifications, significantly influence the behavior of NK cells within the TME [18]. By downregulating activating ligands on tumor cells, these alterations facilitate immune evasion and impair NK cell recognition. Epigenetic drugs, such as HDACis and DNMTis, offer the potential to reprogram the immune landscape and restore anti-tumor activity [58, 59]. In this context, combining epigenetic therapy with NK cell immunotherapy emerges as a promising strategy to overcome resistance and improve therapeutic outcomes [31]. Understanding the interplay between epigenetics and immunity is essential for developing innovative, personalized cancer immunotherapies that enhance clinical efficacy [18].

**FIGURE 2:**
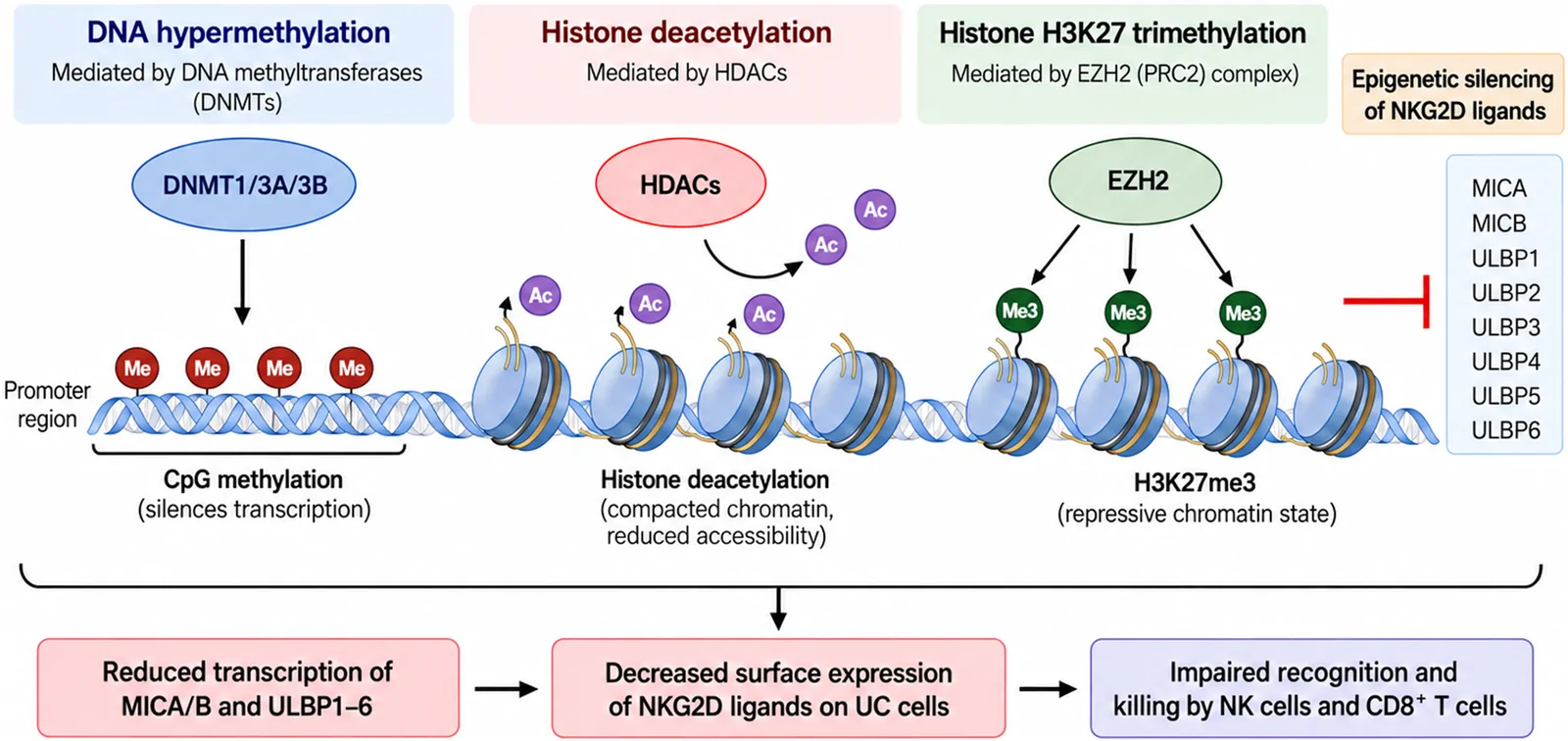
Epigenetic reprogramming restores NK-cell antitumor immunity in urothelial carcinoma. DNA hypermethylation and histone deacetylation reduce the expression of NKG2D ligands, including MICA/B and ULBPs, on urothelial carcinoma cells, thereby impairing NKG2D-mediated NK-cell activation. Reduced ligand engagement attenuates DAP10-associated downstream signaling through PI3K and Grb2-Vav1, resulting in impaired cytoskeletal reorganization, lytic granule polarization, and exocytosis, with reduced perforin, granzyme B, and IFN-γ responses. Treatment with DNA methyltransferase inhibitors (DNMTis) or histone deacetylase inhibitors (HDACis) restores NKG2D ligand expression and reactivates NKG2D-DAP10 signaling, promoting cytoskeletal reorganization, lytic granule polarization and exocytosis, and enhanced NK-cell cytotoxicity, thereby restoring antitumor immunity.
